# Identification of the Potential Functions of the *PKA-R1* Gene in the Regulation of Growth Performance and Molting of *Macrobrachium nipponense* by RNAi

**DOI:** 10.3390/ijms27146139

**Published:** 2026-07-09

**Authors:** Yuefan Zhang, Wenyi Zhang, Yiwei Xiong, Hui Qiao, Hongtuo Fu, Sufei Jiang, Shubo Jin

**Affiliations:** 1 Key Laboratory of Mariculture & Stock Enhancement in North China’s Sea, Ministry of Agriculture and Rural Affairs, Dalian Ocean University, Dalian 116023, China; 13377712674@163.com (Y.Z.); qiaoh@ffrc.cn (H.Q.); 2 Key Laboratory of Freshwater Fisheries and Germplasm Resources Utilization, Ministry of Agriculture and Rural Affairs, Freshwater Fisheries Research Center, Chinese Academy of Fishery Sciences, Wuxi 214081, China; zhangwy@ffrc.cn (W.Z.); xiongyw@ffrc.cn (Y.X.); fuht@ffrc.cn (H.F.); 3 Wuxi Fisheries College, Nanjing Agricultural University, Wuxi 214081, China

**Keywords:** *Macrobrachium nipponense*, *PKA-R1*, growth, molting frequency, RNA interference

## Abstract

*Macrobrachium nipponense* holds significant economic importance in freshwater aquaculture in China, where larger body weight and longer body length are directly associated with higher market demand and improved commercial value. Consequently, identifying genes associated with growth traits is a priority for enhancing economic returns. Previous studies have predicted that the cAMP-dependent protein kinase type I regulatory subunit-like (*PKA-R1*) may be involved in the growth regulation of *M. nipponense*. In this study, we functionally characterized the *Mn-PKA-R1* gene to investigate its potential involvement in growth and molting regulation in *M. nipponense* through qPCR analysis and RNA interference (RNAi). The open reading frame of *Mn-PKA-R1* spanned 3345 base pairs, encoding a protein of 1114 amino acids. The *Mn-PKA-R1* amino acid sequence showed the highest identity with that of *Macrobrachium rosenbergii*, followed by *Penaeus vannamei*. Tissue distribution analysis revealed that *Mn-PKA-R1* was ubiquitously expressed across all examined tissues, with the highest transcript level detected in the testis, suggesting a potential association with testis-related physiological functions. Functional validation via RNA interference showed that repeated *dsPKA-R1* treatment was associated with reduced molting frequency and relative body weight compared with the non-targeting *dsRNA* control group. From day 14 to day 42, the relative body weight compared with day 0 in the non-targeting *dsRNA* control group was significantly higher than that in the *dsPKA-R1*-injected group. Additionally, the molting frequency in the non-targeting *dsRNA* control group was significantly higher than that in the *dsPKA-R1*-injected group at days 7, 14, 21, 28, and 35. These findings suggest that *Mn-PKA-R1* may participate in the regulation of growth and molting in *M. nipponense* under the tested experimental conditions. This study provides preliminary functional evidence for the involvement of *Mn-PKA-R1* in growth-related traits in *M. nipponense*.

## 1. Introduction

The oriental river prawn, *Macrobrachium nipponense*, is widely distributed across China and other Asian countries [1,2]. In China, this species produces about 230,000 metric tons annually, representing 5.72% of total freshwater prawn production [3]. It occupies a prominent position in China’s freshwater aquaculture industry and has become one of the country’s major cultured prawn species. *M. nipponense* exhibits several favorable biological characteristics, including rapid growth, strong adaptability to diverse environmental conditions, high fecundity, and robust disease resistance. These attributes make it particularly suitable for large-scale aquaculture and underscore its considerable economic significance [4].

Growth traits are among the primary targets for genetic improvement in aquatic animals because of their close association with production efficiency and economic value in aquaculture. In general, larger individuals command higher market prices and produce greater economic returns than smaller ones, making growth performance a major concern in aquaculture breeding. Consequently, the improvement of growth-related traits has consistently been regarded as one of the central objectives of breeding programs for most cultured species. It is well established that traits such as body weight and body length are complex quantitative traits controlled by multiple genes, each contributing a relatively small effect. Owing to their moderate heritability and direct impact on production performance, these traits are of considerable importance in selective breeding and have become a major focus of genetic improvement in aquaculture species [5]. Furthermore, growth traits are key determinants of aquaculture production cycles, product quality, and the extent of sexual dimorphism. Variation in growth performance can directly affect the duration of the culture period, the final market size of cultured animals, and the overall commercial value of aquaculture products. In many aquatic species, differences in growth traits are also closely associated with sex-related phenotypic variation, thereby further influencing production efficiency and breeding strategies [6,7,8]. Improvement of growth traits through selective breeding is heritable and can generate sustained long-term benefits for aquaculture. Therefore, there is an urgent need to elucidate the genetic basis of growth performance in this species, including the identification of growth-related genes and associated single-nucleotide polymorphisms (SNPs).

In recent years, studies on the growth performance of *M. nipponense* have covered multiple environmental and physiological factors, including nutrition, temperature, salinity, molting [9], and environmental adaptation [10,11]. Genome-wide association study (GWAS) has, at the genetic level, provided the first evidence that growth traits in *M. nipponense* have a strong hereditary basis, with SNP loci associated with body weight, body length, and other growth-related traits identified on multiple chromosomes. At the same time, a significant genetic correlation between sex and growth has also been detected, indicating that the growth advantage observed in *M. nipponense* is closely associated with sex [12]. In addition, GWAS and comparative transcriptomic association analyses were performed to screen for candidate genes related to growth traits in fast- and slow-growing *M. nipponense* [13]. Among the candidates identified, the *ACTL* gene was selected, and its association with growth was subsequently validated through RNA interference (RNAi) and SNP association analyses [14].

Recently, *Mn-PKA-R1* was identified as a candidate gene associated with growth performance through a GWAS of *M. nipponense*. A previous study identified two growth-related SNPs located in the *Mn-PKA-R1* gene (*p* = 1.5618 × 10^−6^), suggesting that this gene may be involved in the growth process of *M. nipponense* and may potentially influence body weight gain or growth rate [13]. The cAMP-dependent protein kinase type I regulatory subunit-like (*PKA-R1*) is a regulatory subunit of protein kinase A (PKA) that plays a pivotal role in the PKA signaling pathway [15]. The PKA signaling pathway is a classical cAMP-dependent signaling cascade that transduces extracellular signals into intracellular responses through phosphorylation of downstream target proteins. The functional specificity of this pathway is achieved through differential expression of PKA subunits, isozyme composition, and subcellular localization mediated by anchoring proteins. It plays essential roles in the regulation of metabolism, gene transcription, cell growth and differentiation, ion channel activity, neural signaling, and reproductive processes [16]. PKA is a heterotetramer consisting of two regulatory subunits and two catalytic subunits. The regulatory subunits contain cAMP-binding domains and interact with the catalytic subunits to modulate PKA activity. When intracellular cAMP levels rise, cAMP binds to the regulatory subunits, thereby triggering the dissociation and activation of the catalytic subunits. In contrast, when the regulatory and catalytic subunits remain associated, PKA is maintained in an inactive state [17,18,19]. It primarily mediates cellular responses to extracellular signals, including hormones, neurotransmitters, and light. When intracellular cAMP levels increase, cAMP binds to the regulatory subunits, leading to their dissociation from the catalytic subunits and thereby activating the kinase. PKA plays a central role in the regulation of the oocyte cell cycle, particularly in meiotic arrest, meiotic resumption, and oocyte maturation, and its function depends on the precise control of both its activity and subcellular localization [20]. As a key mediator of intracellular signaling, activated PKA exerts its regulatory functions by phosphorylating multiple substrate proteins [21,22], thereby regulating a wide range of cellular processes across diverse tissue types, including gene transcription, energy metabolism, cell proliferation and differentiation, and programmed cell death [23,24,25,26].

In the present study, we investigated the potential role of *Mn-PKA-R1* in regulating growth performance in *M. nipponense* using quantitative PCR (qPCR) and RNAi. Our findings are expected to provide preliminary functional evidence for the involvement of *Mn-PKA-R1* in growth and molting regulation in *M. nipponense* and provide a basis for further investigation of growth-related functional genes in this species.

## 2. Results

### 2.1. Sequence Analysis

**Figure 1:**
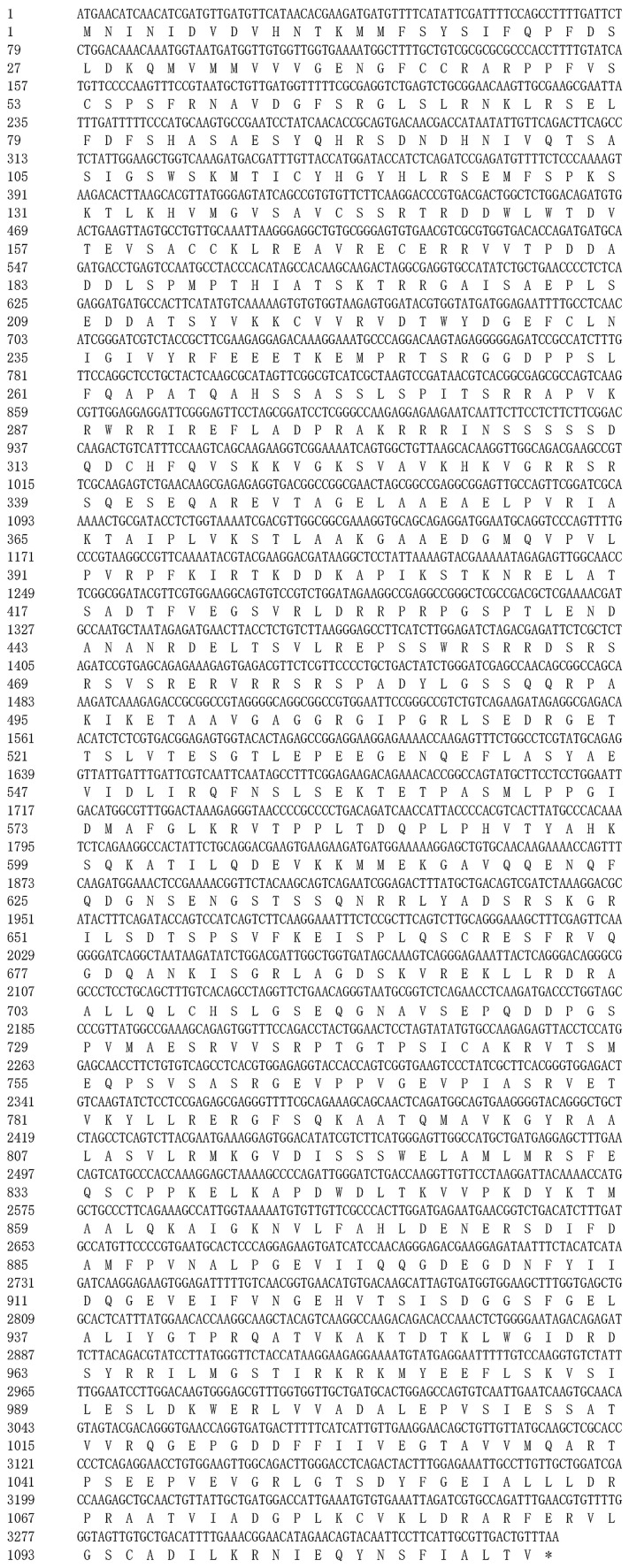
The open reading frame sequence of the *Mn-PKA-R1* gene. The nucleotide sequence and the deduced amino acid sequence are presented from 5′ to 3′. Amino acids in the deduced sequence are represented by single uppercase letters. The initiation codon (ATG) and the termination codon (TAA, indicated by an asterisk) are clearly marked.

The open reading frame of *Mn-PKA-R1* spanned 3345 base pairs, encoding a protein of 1114 amino acids. The gene was positioned between nucleotide coordinates 128,346,744 and 128,443,339 on chromosome 4 (Figure 1). The predicted molecular weight of the *Mn-PKA-R1* protein was 123.51 kDa, and its theoretical isoelectric point was 8.726.

### 2.2. Multiple Sequence Alignment and Phylogenetic Tree Analysis

**Figure 2:**
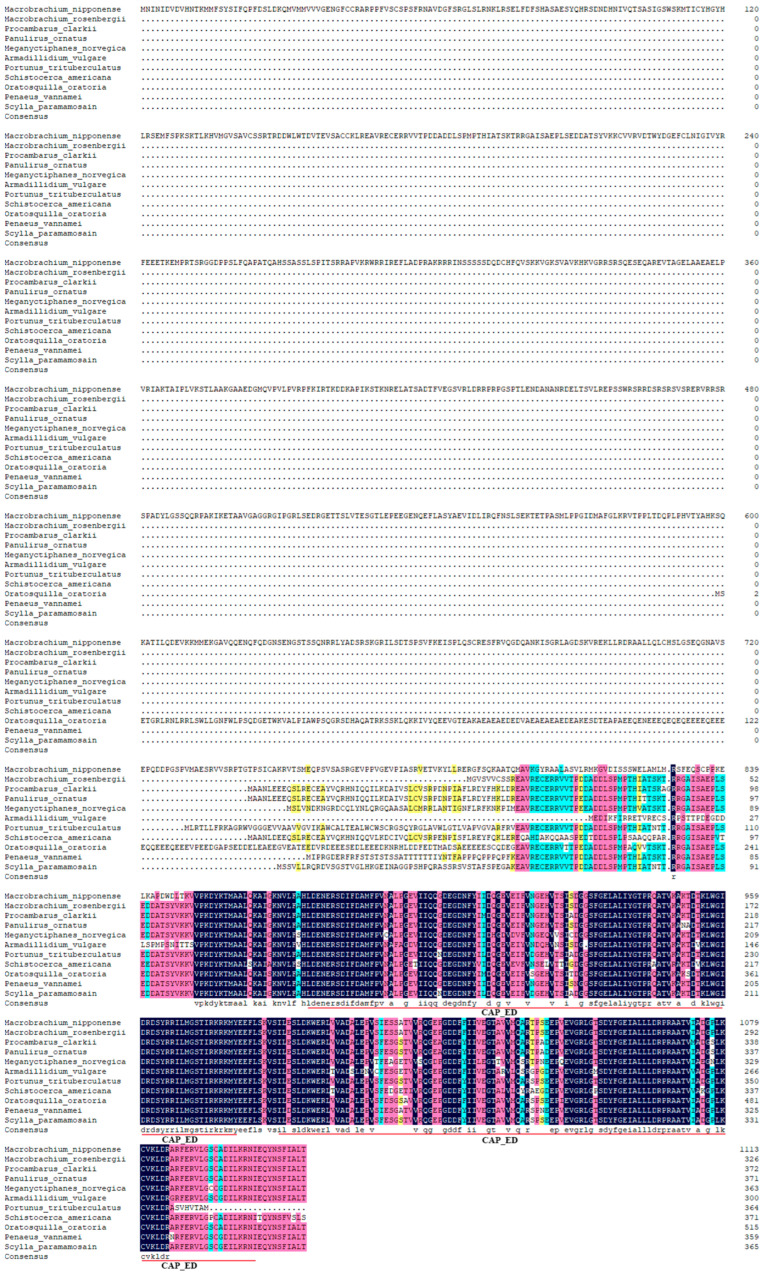
Amino acid sequence alignment of *PKA-R1* from *M. nipponense* and other species is shown. Identical amino acids are indicated in black, and the numbers on the right denote the amino acid positions of *PKA-R1* in different species. Different colors represent the degree of homology level. Black indicates 100% identity between species, pink indicates ≥75% identity, blue indicates ≥50% identity, and yellow indicates ≥33% identity.

**Figure 3:**
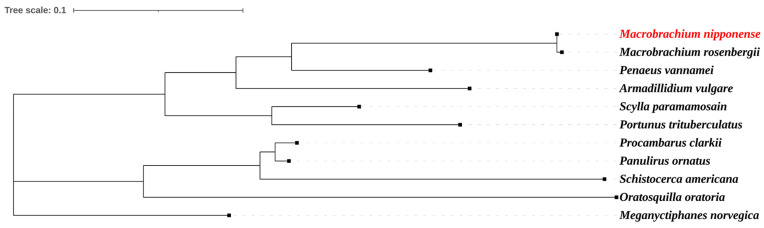
Phylogenetic tree of *PKA-R1*. *M. nipponense* is highlighted in red.

Multiple sequence alignment (Figure 2) showed that *Mn-PKA-R1* exhibited a high degree of amino acid sequence identity with homologs from other identified species, ranging from 85.45% to 99.25%. The *Mn-PKA-R1* amino acid sequence showed the highest identity with that of *Macrobrachium rosenbergii* (99.25%), followed by *Penaeus vannamei* (96.99%), whereas the lowest identity was observed with *Schistocerca americana* (85.45%). Domain prediction analysis revealed a conserved CAP_ED domain at amino acid residues 870–979 and 988–1103, annotated as the effector domain of CAP family transcription factors. The neighbor-joining phylogenetic tree based on *Mn-PKA-R1* amino acid sequences (Figure 3) clustered *M. nipponense*, *M. rosenbergii*, and *P. vannamei* into a conserved crustacean clade, suggesting that *Mn-PKA-R1* is closely related among crustacean species.

### 2.3. qPCR Analysis in Different Mature Tissues

**Figure 4:**
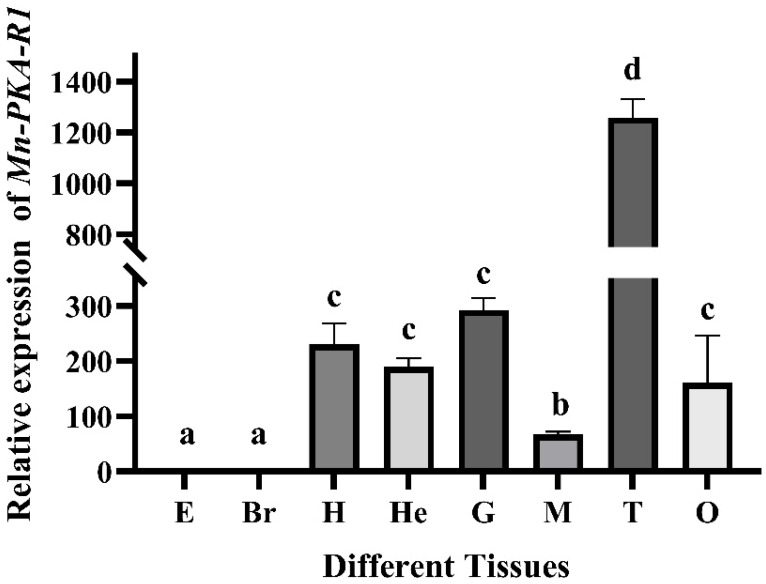
Relative expression levels of *Mn-PKA-R1* in different tissues of *M. nipponense*. The *EIF* gene served as the internal reference for normalization. Data are expressed as the mean ± standard deviation (SD; *n* = 6). E, eyestalk; Br, brain; H, heart; He, hepatopancreas; G, gill; M, muscle; O, ovary; T, testis. Different lowercase letters (a–d) above the bars indicate significant differences among different tissues (*p* < 0.05); bars sharing the same letter are not significantly different.

The qPCR analysis indicated that *Mn-PKA-R1* mRNA was ubiquitously expressed in all examined tissues (Figure 4). Among these tissues, the highest expression level was detected in the testis, which showed a statistically significant difference compared with other tissues (*p* < 0.05), whereas the lowest expression was observed in the eyestalk. The expression level of this gene was 1257-fold higher in the testis than that in the eyestalk.

### 2.4. RNAi Efficiency Verification

**Figure 5:**
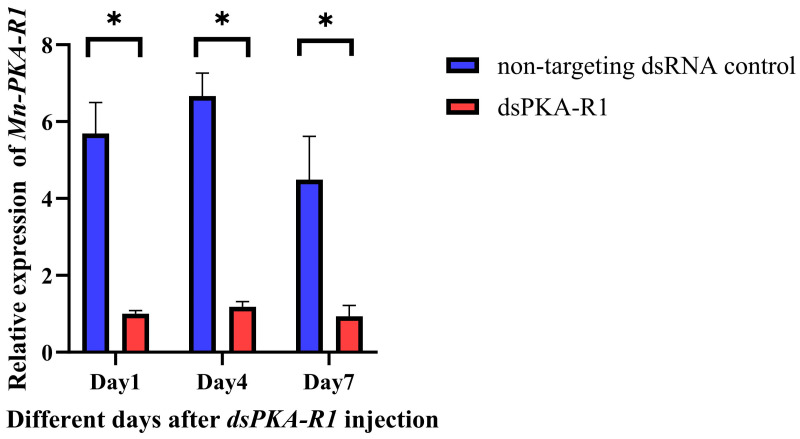
Relative expression levels of *Mn-PKA-R1* in muscle tissue following dsRNA injection. * indicates a significant difference (*p* < 0.05) in *Mn-PKA-R1* expression between the *dsGFP*-injected group and the *dsPKA-R1*-injected group.

The silencing efficiency of the synthesized *dsPKA-R1* was evaluated by qPCR at 1, 4, and 7 days after *dsPKA-R1* injection (Figure 5). The qPCR results showed that *Mn-PKA-R1* mRNA expression in muscle tissue was significantly reduced in the *dsPKA-R1*-injected group compared with the non-targeting *dsRNA* control group. Specifically, *Mn-PKA-R1* expression decreased by 82.39%, 82.23%, and 79.19% at 1, 4, and 7 days post-injection, respectively, and all reductions were statistically significant (*p* < 0.05).

### 2.5. Functional Analysis of Mn-PKA-R1 in Growth

**Figure 6:**
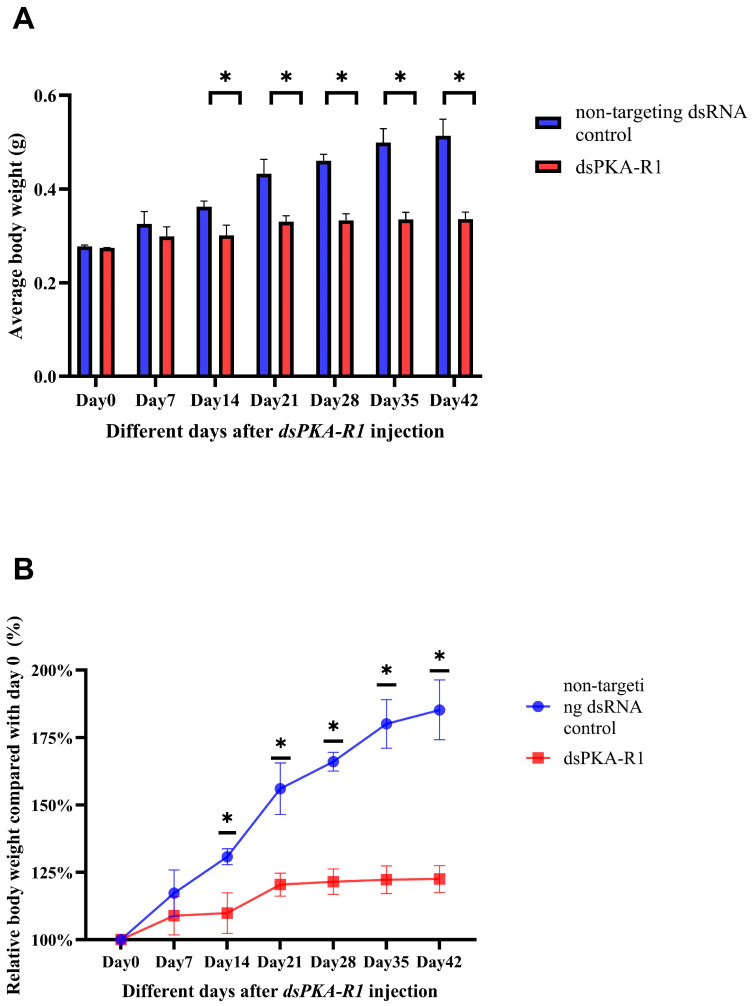
Effects of *dsPKA-R1* injection on body weight in *M. nipponense*. Body weight was monitored over time after injection with *dsPKA-R1* or *dsGFP*. The *dsGFP*-injected group served as a non-targeting *dsRNA* injection control. Significant differences between the *dsPKA-R1*-injected group and the *dsGFP*-injected non-targeting *dsRNA* control group at the same time points are indicated by * (*p* < 0.05). (**A**) Body weight change in *M. nipponense*. (**B**) Relative body weight compared with day 0 in *M. nipponense*. Relative body weight compared with day 0 was calculated as body weight at each time point/initial body weight × 100%.

In the long-term interference experiment, prawns with similar initial body weights of approximately 0.27 g were selected for RNAi analysis. The initial mean body weights of prawns at day 0 were comparable between the non-targeting *dsRNA* control group (0.277 ± 0.003 g) and the *dsPKA-R1*-injected group (0.274 ± 0.001 g). Over the course of the experiment, body weight in the non-targeting *dsRNA* control group increased steadily, reaching 0.325 ± 0.027 g, 0.362 ± 0.012 g, 0.432 ± 0.031 g, 0.460 ± 0.014 g, 0.499 ± 0.030 g, and 0.513 ± 0.036 g at days 7, 14, 21, 28, 35, and 42, respectively. In contrast, prawns in the *dsPKA-R1*-injected group showed markedly lower body weights at the corresponding time points, measuring 0.298 ± 0.021 g, 0.301 ± 0.022 g, 0.330 ± 0.013 g, 0.333 ± 0.014 g, 0.335 ± 0.015 g, and 0.334 ± 0.015 g, respectively (Figure 6A). Statistical analysis showed that body weights in the *dsPKA-R1*-injected group were significantly lower than those in the non-targeting *dsRNA* control group from day 14 onward (*p* < 0.05), indicating that *dsPKA-R1* injection was associated with reduced body weight increase during the experimental period. Relative body weight compared with day 0 was calculated as body weight at each time point/initial body weight × 100%. Compared with the initial body weight, the relative body weight of *M. nipponense* in the non-targeting *dsRNA* control group reached 117.27%, 130.79%, 156.01%, 166.04%, 180.09%, and 185.24% at days 7, 14, 21, 28, 35, and 42, respectively. In the *dsPKA-R1*-injected group, the corresponding relative body weight values were 108.87%, 109.84%, 120.43%, 121.52%, 122.25%, and 122.49%, respectively. From day 14 onward, the relative body weight compared with day 0 in the non-targeting *dsRNA* control group was significantly higher than that in the *dsPKA-R1*-injected group (*p* < 0.05; Figure 6B).

### 2.6. Functional Analysis of Mn-PKA-R1 in Molting

Throughout the experimental period, the molting frequency in the *dsPKA-R1* group remained consistently lower than that in the non-targeting *dsRNA* control group at all examined time points. Specifically, the mean molting frequencies in the non-targeting *dsRNA* control group at Day 7, Day 14, Day 21, Day 28, Day 35, and Day 42 were 8.67 ± 1.53, 6.67 ± 1.53, 6.67 ± 2.31, 5.33 ± 0.58, 3.67 ± 1.15, and 1.67 ± 0.58, respectively, whereas the corresponding values in the *dsPKA-R1* group were 5.00 ± 1.00, 2.33 ± 1.15, 4.00 ± 1.00, 2.33 ± 0.58, 0.33 ± 0.58, and 0.33 ± 0.58.

**Figure 7:**
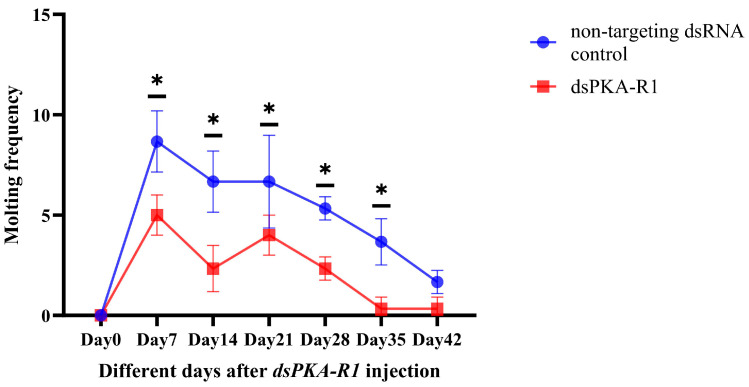
Effects of *dsPKA-R1* injection on molting frequency in *M. nipponense*. The molting frequency of *M. nipponense* was monitored in the non-targeting *dsRNA* control group and the *dsPKA-R1*-injected group from Day 0 to Day 42 after injection. Significant differences between the *dsPKA-R1*-injected group and the non-targeting *dsRNA* control group at the same time points are indicated by * (*p* < 0.05).

Statistical analysis showed that the molting frequency in the *dsPKA-R1* group was significantly lower than that in the non-targeting *dsRNA* control group at Day 7, Day 14, Day 21, Day 28, and Day 35 (*p* < 0.05). By Day 42, although the molting frequency in the *dsPKA-R1* group remained lower than that in the non-targeting *dsRNA* control group, the difference was no longer statistically significant (Figure 7). In addition, cumulative molting counts revealed that a total of 98 molts were recorded in the non-targeting *dsRNA* control group during the experimental period, whereas only 43 molts were observed in the *dsPKA-R1* group, representing 43.9% of the non-targeting *dsRNA* control level. These results suggest that *Mn-PKA-R1* knockdown was associated with reduced molting frequency in *M. nipponense* under the tested conditions.

## 3. Discussion

Growth-related traits are major targets in the genetic improvement of *M. nipponense* as they are closely linked to production performance and economic value [7,27,28]. Growth traits in *M. nipponense* are regulated by multiple factors, including temperature [29], feed composition [30], feeding strategy [31] and gene expression [32]. Studies investigating the role of gene expression in growth regulation are still relatively limited. Therefore, investigating genes associated with growth traits in *M. nipponense* is of considerable importance. Based on the GWAS results, *Mn-PKA-R1* has been identified as a potential candidate gene related to growth traits [13]. Accordingly, the present study investigated the potential involvement of *Mn-PKA-R1* in the growth performance, providing a basis for the genetic improvement of growth traits in this species.

Research on the role of *PKA-R1* in the growth and developmental processes of aquatic animals remains limited. In the silkworm, PKA has been reported to be involved in a variety of cellular processes, including growth, proliferation, and differentiation. Moreover, PKA activity has been found to be negatively correlated with parthenogenetic efficiency, with excessive PKA activation delaying embryonic development and PKA inhibition disrupting cell cycle progression [25]. PKA facilitates sperm capacitation through the regulation of ion channels and enzyme activity. To acquire fertilization competence, mammalian sperm must undergo a process known as capacitation, which is dependent on the early activation of protein kinases [33]. These studies suggest that PKA signaling may be involved in developmental and reproduction-related physiological processes in animals. In the present study, qPCR analysis showed that *Mn-PKA-R1* was expressed in all examined tissues, with the highest expression detected in the testis, suggesting a potential association with testis-related physiological functions. However, because no gonadal histology, reproductive phenotype analysis, spermatogenesis-related assays, or reproduction-related gene expression analysis was performed in the present study, the high expression of *Mn-PKA-R1* in the testis should not be interpreted as direct evidence that *Mn-PKA-R1* regulates testicular development or reproductive function. Previous studies have demonstrated that gonadal development is closely linked to growth. Gonadal development is typically accompanied by pronounced energy reallocation. Limited nutrients and energy reserves are preferentially invested in gametogenesis, vitellogenesis, and other reproduction-related processes, thereby reducing the energy available for somatic growth. As a result, growth rate may decline, body weight gain may be constrained, and stored energy may be redirected from somatic tissues to the gonads [34,35]. Nevertheless, gonadal status is positively associated with somatic growth in *Oreochromis mossambicus*; intact gonads exert a promoting effect on body growth, whereas gonad ablation suppresses growth, and ectopic gonad transplantation fully rescues the growth impairment [36]. In *M. nipponense*, these two processes are not regulated independently, but instead may be linked during first sexual maturity through sex-specific patterns of resource allocation and partially overlapping genetic or endocrine mechanisms [37,38]. Therefore, the high testis expression of *Mn-PKA-R1* is noteworthy, but its possible role in reproductive development or sex-specific growth regulation requires further investigation.

RNAi is initiated by double-stranded RNA (dsRNA), which is processed by Dicer into small interfering RNAs (siRNAs) of approximately 21–22 nucleotides. These siRNAs are then incorporated into the RNA-induced silencing complex (RISC). During RISC activation, the passenger strand is removed, whereas the guide strand directs sequence-specific binding to the target mRNA. The target transcript is subsequently cleaved and degraded, resulting in post-transcriptional gene silencing and reduced mRNA abundance [39]. In *M. nipponense*, RNAi technology is well established, and numerous genes have been successfully knocked down in this species [40,41,42,43]. However, the functional involvement of *PKA-R1* in growth performance and molting regulation in *M. nipponense* has not been experimentally validated. Therefore, the present study used RNAi to provide preliminary functional evidence for the potential role of *PKA-R1* in these processes. qPCR analysis revealed that *Mn-PKA-R1* expression levels were significantly reduced following *dsPKA-R1* injection compared to those in the non-targeting *dsRNA* control group on the same day, thereby confirming the efficacy of the synthesized dsRNA within 7 days after injection. During the 42-day RNAi experiment, *dsRNA* injection and phenotypic measurement were performed every 7 days to maintain the RNAi treatment. The relative body weight compared with day 0 of prawns in the non-targeting *dsRNA* control group was higher than that of prawns in the *dsPKA-R1*-injected group from day 14 onward. These results indicate that repeated *dsPKA-R1* treatment was associated with reduced growth performance compared with the non-targeting *dsRNA* control group under the tested experimental conditions. Together, these findings provide preliminary functional evidence that *Mn-PKA-R1* may participate in the regulation of growth performance in *M. nipponense*, although the underlying mechanism requires further investigation.

Previous studies have reported that individual growth may be regulated by the molting frequency [32]. Individuals with a higher molting frequency tended to exhibit faster growth. Accordingly, we further examined the potential involvement of *Mn-PKA-R1* in molting. The cumulative molting count in the non-targeting *dsRNA* control group was 2.28-fold higher than that in the *dsPKA-R1* group, suggesting that *Mn-PKA-R1* may also be involved in the molting process in *M. nipponense* under the tested experimental conditions. Considering that molting is closely associated with growth in crustaceans, the reduced molting frequency observed after repeated *dsPKA-R1* treatment may partly explain the lower growth performance in the *dsPKA-R1*-injected group. However, the detailed mechanism linking *Mn-PKA-R1*, molting, and growth requires further investigation.

## 4. Materials and Methods

### 4.1. Tissue Collection

In total, 240 healthy individuals of *M. nipponense* were obtained from the Dapu Breeding Base in Wuxi, China (120°13′44″ E, 31°28′22″ N) (Table 1). All prawns were acclimated for 3 days under controlled laboratory conditions prior to the experiment, during which the water temperature was maintained at 26.0 ± 1.2 °C and dissolved oxygen was kept above 6.0 mg/L.

Muscle tissue was dissected from 10 randomly selected individuals to verify the ORF sequence of *Mn-PKA-R1*. For qPCR analysis, 30 healthy *M. nipponense* were collected, and tissues including the eyestalk, brain, heart, hepatopancreas, gill, muscle, ovary, and testis were sampled. Tissues from five individuals were pooled as one biological replicate, and six biological replicates were prepared for each tissue. For RNAi analysis, 200 prawns with similar initial body weights of approximately 0.27 g were used. Because sex could not be accurately determined at this stage, the individuals were not separated by sex during the RNAi experiment. After injection with double-stranded *GFP* (*dsGFP*) or double-stranded *PKA-R1* (*dsPKA-R1*), muscle tissue was collected for qPCR analysis.

### 4.2. Annotation and Comparison of Mn-PKA-R1

The full-length cDNA sequence of *Mn-PKA-R1* was obtained from the *M. nipponense* genome database (accession number: GCA_015104395.2).

To verify the sequence, total RNA was extracted from each muscle sample using RNAiso Plus reagent (TaKaRa, Dalian, China). RNA concentration was measured with a spectrophotometer (Eppendorf, Hamburg, Germany), and RNA integrity was assessed by 1.2% agarose gel electrophoresis. Approximately 1 μg of total RNA from each sample was reverse-transcribed to synthesize first-strand cDNA using the iScript™ cDNA Synthesis Kit (Bio-Rad, Hercules, CA, USA). Primers were designed using Primer 5.0 software, and subsequent experimental procedures were carried out according to previously described methods [44]. To confirm sequence fidelity, we validated the obtained sequence experimentally using four specific primer pairs (Table 2) and the synthesized cDNA as template. The PCR products were then sequenced by Shanghai Shenggong Bioengineering Technology Service Co., Ltd. (Shanghai, China) on an ABI 3730 automated DNA sequencer (Invitrogen Biotechnology Co., Ltd., Carlsbad, CA, USA).

The ORF of *Mn-PKA-R1* was predicted using the online ORF-FINDER tool (https://www.ncbi.nlm.nih.gov/orffinder, 22 December 2025) [45]. The corresponding cDNA sequence was translated into the amino acid sequence and analyzed using DNAman software (version 9.0) [46]. The molecular weight (MW) and theoretical isoelectric point (pI) of the predicted *Mn-PKA-R1* amino acid sequence were determined using the Protein_iep tool available from Novopro (https://www.novopro.cn/tools/protein_iep.html, 23 December 2025) [47]. Similarity analysis of the *Mn-PKA-R1* amino acid sequence with other *PKA-R1* sequences was conducted using NCBI BLASTp (https://blast.ncbi.nlm.nih.gov/Blast.cgi, 23 December 2025, version 2.17.0.). Multiple sequence alignment was performed with DNAMAN software (version 9.0). A phylogenetic tree was constructed using the neighbor-joining (NJ) method with 1000 bootstrap replications in MEGA software (version 11.0) [48]. The *PKA-R1* amino acid sequences included in the phylogenetic analysis are provided in Table 3.

### 4.3. qPCR Analysis

qPCR was conducted to determine the mRNA expression level of *Mn-PKA-R1*. Total RNA was extracted from each tissue sample using RNAiso Plus Reagent (TaKaRa) in accordance with the manufacturer’s protocol. RNA concentration was measured with a spectrophotometer (Eppendorf, Hamburg, Germany), and RNA integrity was examined by agarose gel electrophoresis. Approximately 1 µg of total RNA from each sample was reverse-transcribed to synthesize first-strand cDNA using the iScript™ cDNA Synthesis Kit (Bio-Rad).

qPCR was carried out following previously established protocols using the UltraSYBR Mixture (CWBIO, Beijing, China) on a Bio-Rad iCycler iQ5 real-time PCR system (Bio-Rad, Hercules, CA, USA) [49]. The PCR thermal cycling conditions consisted of an initial denaturation at 95 °C for 30 s, followed by 40 cycles of 95 °C for 10 s, 60 °C for 10 s, and 72 °C for 30 s. Eukaryotic translation initiation factor 5A (*EIF*) was selected as the internal reference gene, as it has been demonstrated to be a reliable reference for qPCR analysis in *M. nipponense* [49]. The specific primers used for qPCR are presented in Table 2. The relative mRNA expression levels of *Mn-PKA-R1* were calculated using the 2^−ΔΔCT^ method [50].

### 4.4. RNAi Analysis

To verify the function of *Mn-PKA-R1*, an RNAi experiment was performed. A total of 200 *M. nipponense* with similar initial body weights of approximately 0.27 g were selected and randomly allocated to two groups, with three replicates per group (approximately 33–34 individuals per replicate); one group was injected with *dsPKA-R1*, whereas the other received *dsGFP*. *dsGFP* was used as a non-targeting *dsRNA* injection control to account for potential effects associated with *dsRNA* injection and handling procedures.

RNAi analysis was conducted to investigate the potential role of *Mn-PKA-R1* in the regulation of growth performance and molting in *M. nipponense*. Specific primers containing the T7 promoter sequence were designed using the online software Snap Dragon (https://www.flyrnai.org/cgi-bin/RNAi_find_primers.pl, 24 December 2025). The dsRNA of *Mn-PKA-R1* (*dsPKA-R1*) was generated using the Transcript Aid™ T7 High Yield Transcription Kit (Fermentas, Inc., Rockville, MD, USA), while the dsRNA of *dsGFP* served as the non-targeting *dsRNA* control. The integrity of the dsRNA was verified by 1.2% agarose gel electrophoresis, and the concentration was determined using a spectrophotometer (Eppendorf, Hamburg, Germany). The synthesized *dsPKA-R1* and *dsGFP* were subsequently preserved at −80 °C. Following established methods [51], prawns in the experimental and non-targeting *dsRNA* control groups were injected with *Mn-PKA-R1* dsRNA and *GFP* dsRNA (both prepared at 4 µg/µL in isotonic solution), respectively, at a dose of 4 µg per gram of body weight. Accordingly, the injection volume administered to each prawn (µL) was equal to its body weight (g). During the 42-day RNAi experiment, prawns were injected with *dsPKA-R1* or *dsGFP* every 7 days. Body weight and molting events were recorded before each injection at days 0, 7, 14, 21, 28, 35, and 42. To assess the efficiency of RNAi, the mRNA expression level of *Mn-PKA-R1* in muscle tissue was determined by qPCR at 1, 4, and 7 days after injection (N ≥ 3 for each time point). Relative body weight compared with day 0 was calculated as body weight at each sampling time/initial body weight at day 0 × 100%. Molting frequency was calculated as the number of molts recorded in each replicate during each 7-day observation interval. The replicate was used as the statistical unit for molting-frequency analysis.

### 4.5. Statistical Analysis

Statistical analyses were performed using SPSS Statistics (version 27.0). For tissue-expression and RNAi-efficiency analyses, significant differences among groups were analyzed using one-way ANOVA followed by appropriate post hoc multiple comparison tests. For body weight, relative body weight compared with day 0, and molting-frequency data, comparisons between the *dsPKA-R1*-injected group and the non-targeting *dsRNA* control group were performed separately at each sampling time point using one-way ANOVA. Quantitative data are presented as the mean ± standard deviation (SD), and a *p*-value of less than 0.05 was regarded as statistically significant.

## 5. Conclusions

This study identified *Mn-PKA-R1* as a candidate growth-related gene in *M. nipponense* and provided preliminary functional evidence for its potential involvement in growth and molting regulation. *Mn-PKA-R1* was expressed in multiple tissues, with the highest expression detected in the testis, suggesting a potential association with testis-related physiological functions. Compared with the non-targeting *dsRNA* control group, repeated *dsPKA-R1* treatment was associated with reduced growth performance and molting frequency under the tested experimental conditions. These results suggest that *Mn-PKA-R1* may participate in the regulation of growth and molting in *M. nipponense*.

## Figures and Tables

**Table 1:** Specimens used in this study.

Sampling Date	Animals	Tissue	Purpose
9–13 July 2025	10 specimens	Muscle	ORF verification
9–13 July 2025	30 specimens	Eyestalk, Brain, Heart, Hepatopancreas, Gill, Muscle, Ovary, Testis	qPCR analysis
19 June–30 July 2025	200 specimens with similar initial body weights of approximately 0.27 g	Muscle	RNAi analysis

**Table 2:** Primers used in the present study.

Primer	Sequence	Purpose
PKA-F1	GATGGTTTTTCGCGAGGTCTG	Primers for PCR verification
PKA-R1	GTCACCTCTCTCGCTTGTTCA	
PKA-F2	GGCTGTTAAGCACAAGGTTGG	
PKA-R2	GTCCTGCAGAATAGTGGCCTT	
PKA-F3	TCGGAGAAGACAGAAACACCG	
PKA-R3	TTTAGCTCCTTTGGTGGGCAT	
PKA-F4	CAGTCGGTGAAGTCCCTATCG	
PKA-R4	GTGCGAGCTTGCATAACAACA	
RT-PKA-F	TGTGTGGTAAGAGTGGATACGTG	Primer for qPCR
RT-PKA-R	CTTGGTCCGAAGAAGAGGAAGAA	
EIF-F1	CATGGATGTACCTGTGGTGAAAC	Primer for reference gene
EIF-R1	CTGTCAGCAGAAGGTCCTCATTA	
dsPKA-F	TAATACGACTCACTATAGGGACGTGGAGAGGTACCACCAG	Primer for RNAi
dsPKA-R	TAATACGACTCACTATAGGGCCCCAGAGTTTGGTGTCTGT	
dsGFP-F	GATCACTAATACGACTCACTATAGGGTCCTGGTCGAGCTGGACGG	Primer for RNAi
dsGFP-R	GATCACTAATACGACTCACTATAGGGCGCTTCTCGTTGGGGTCTTTG	

**Table 3:** Species used for the construction of the phylogenetic tree in the present study.

Species	Accession Number
*Macrobrachium nipponense*	PZ344223
*Macrobrachium rosenbergii*	XP_066943473.1
*Penaeus vannamei*	XP_069981577.1
*Oratosquilla oratoria*	XP_076028577.1
*Procambarus clarkii*	XP_045601806.1
*Panulirus ornatus*	XP_071550922.1
*Meganyctiphanes norvegica*	CAL4075166.1
*Armadillidium vulgare*	RXG69763.1
*Portunus trituberculatus*	XP_045133056.1
*Schistocerca americana*	XP_046995590.1
*Scylla paramamosain*	XP_063865395.1

## Data Availability

The original contributions presented in this study are included in the article. Further inquiries can be directed to the corresponding authors.
